# An unusual case of retrovesical ectopic prostate tissue accompanied by primary prostate cancer

**DOI:** 10.1186/1477-7819-10-186

**Published:** 2012-09-11

**Authors:** Fu-Qing Tan, Xin Xu, Bo-Hua Shen, Jie Qin, Ke Sun, Qihan You, De-Sheng Shang, Xiang-Yi Zheng

**Affiliations:** 1 Department of Urology, The First Affiliated Hospital, Zhejiang University School of Medicine, Hangzhou, 310003, China; 2 Department of pathology, The First Affiliated Hospital, Zhejiang University School of Medicine, Hangzhou, China; 3 Department of Radiology, The First Affiliated Hospital, Zhejiang University School of Medicine, Hangzhou, China

**Keywords:** Ectopic prostate, Retrovesical space, Prostate cancer

## Abstract

We report an unusual case of retrovesical ectopic prostate tissue in a 73-year-old man with primary prostate cancer. The man’s prostate-specific antigen was 24.66 ng/ml.Transabdominal ultrasonography, pelvic computed tomography,and pelvic magnetic resonance imaging demonstrated a heterogeneous 8.5 × 8.0 × 7.0 cm mass in contact with the posterior wall of the urinary bladder. The patient underwent a retropubic radical prostatectomy and resection of tumor. Pathological examination of prostate revealed a prostatic adenocarcinoma, Gleason score of 4 + 5 = 9, and the retrovesical tumor was confirmed to be a benign prostate tissue.

## Background

Ectopic prostatic tissue is a relatively uncommon entity that is most commonly encountered in the lower male genitourinary tract [1]. However, only 10 cases of ectopic prostatic tissue situated in the retrovesical space have been published up to date [2-4]. Here, we report an unusual case of retrovesical benign ectopic prostatic tissue accompanied by primary prostate cancer.

## Case report

**Figure 1:**
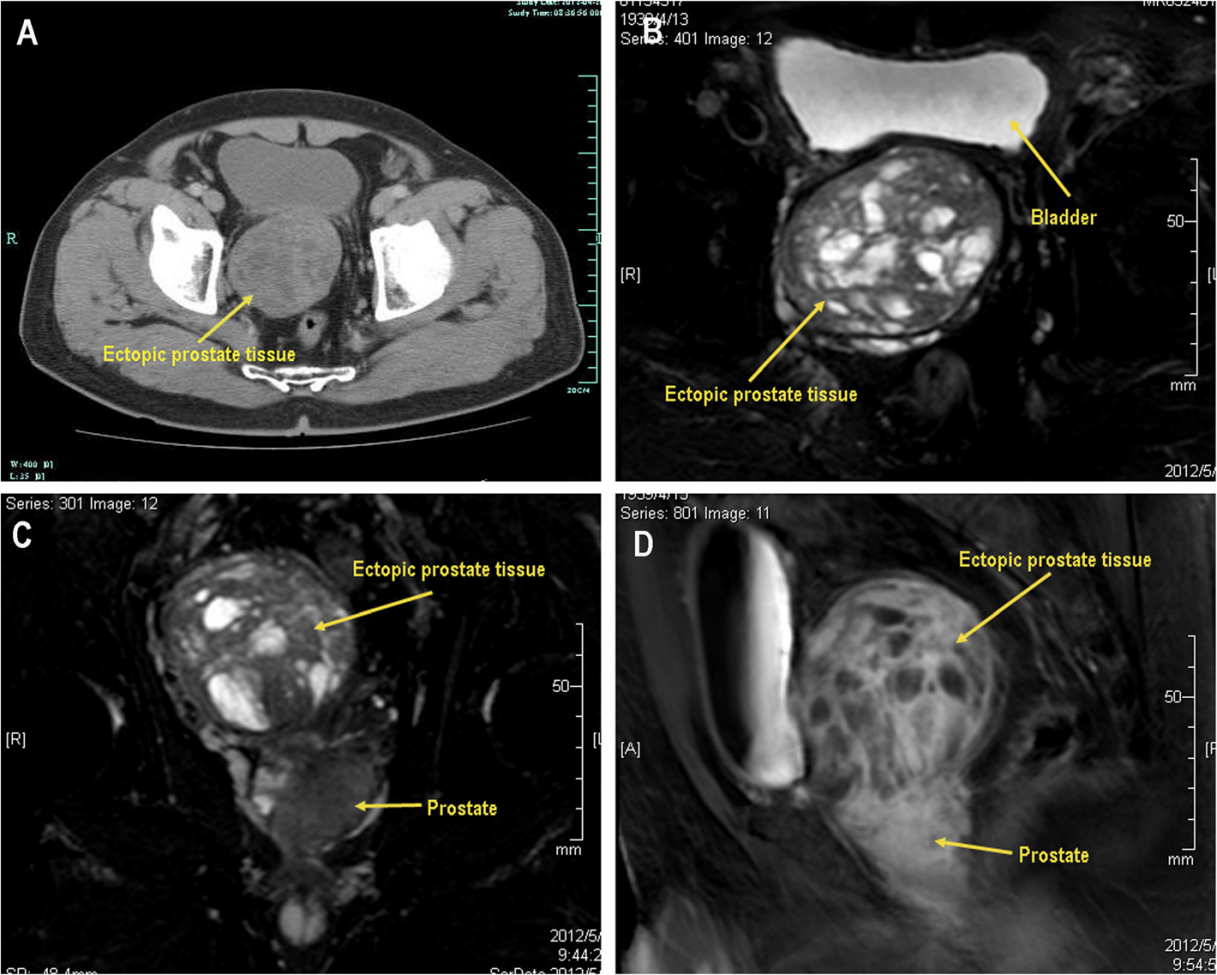
** Pelvic computed tomography (A) demonstrated a heterogeneous contrast-enhanced mass (*arrow*) 8 cm in diameter in the retrovesical space. B**, **C**, **D**: Pelvic magnetic resonance imaging showed a tumor (*arrow*) of heterogeneous intensity with a multilocular cystic structure.

**Figure 2:**
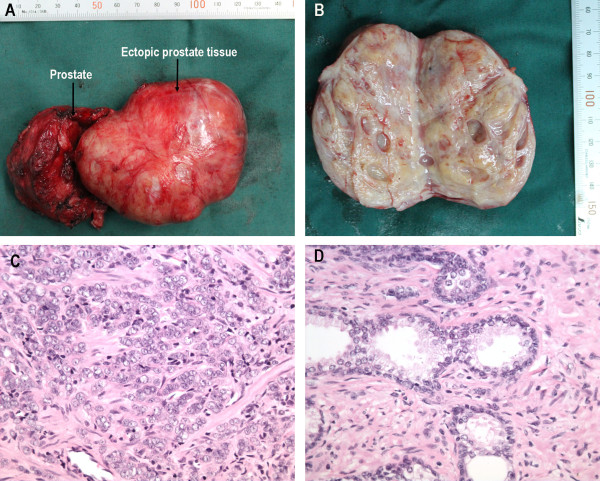
**A: Resected specimens of prostate (*arrow*), seminal vesicle and the tumor (*arrow*) with acomplete capsule. B**: The tumor had a multilocular cystic structure with a gray cut surface. **C**: Section of the prostate revealed a prostatic adenocarcinoma, Gleason score of 4 + 5 = 9. **D**: Section of the tumor showed a benign prostate tissue (**D**). (H&E stain, **C**, **D**: ×400).

A 73-year-old man suffering from progressive dysuria was admitted to the urology department of our hospital. His medical, personal, and family histories were unremarkable. Transabdominal ultrasonography confirmed a heterogeneous tumor with solid and cystic lesions in contact with the posterior wall of the urinary bladder, while urography showed a normal upper urinary tract. Pelvic computed tomography demonstrated a heterogeneous contrast-enhanced mass about 8.0 cm in diameter in the retrovesical space (Figure 1). Pelvic magnetic resonance imaging showed a tumor of heterogeneous intensity with a multilocular cystic structure (Figure 1). The level of the prostate-specific antigen (PSA) showed an elevation (24.66 ng/ml). It was not clear where the retrovesical tumor originated from. A transrectal ultrasound-guided biopsy was subsequently performed, which failed to get the retrovesical tumor but revealed a primary prostatic adenocarcinoma, with Gleason score of 4 + 3 = 7. Following discussion of therapeutic options, the patient underwent a retropubic radical prostatectomy and resection of tumor. The resected tumor was elliptical with complete capsule and measured 8.5 × 8.0 × 7.0 cm in size. At pathological examination, the mass was confirmed to be benign prostatic tissue (Figure 2). Histological diagnosis of the prostate suggested prostate adenocarcinoma, Gleason score of 4 + 5 = 9, involving both sides of the gland (Figure 2). There was no evidence of lymph node metastasis, but seminal vesicle invasion, extraprostatic extension, perineural invasion were confirmed.

## Discussion

Ectopic prostate is an unusual but not uncommon finding in the genitourinary tract [5]. Most aberrant prostatic tissue occurs in the urethra [6] and urinary bladder [7], but has also been observed in the testis [8], epididymis [9], seminal vesicle [5], cervix, and vagina [10]. Up to now, a few cases of retrovesical ectopic prostate have been reported in English medical literature [2-4]. However, to our knowledge, there have been no previously reported cases of retrovesical ectopic prostate accompanied by primary prostate cancer.

The source of ectopic prostatic tissue is not entirely clear, and numerous different theories have been proposed to explain this phenomenon, such as migration and misplacement of normal tissue, persistence of embryonic remnants, and metaplastic change caused by chronic inflammation [11,12]. For aberrant prostatic tissue outside the urinary tract, the main possible interpretation seems to be that the embryonic prostatic tissue migrated and became isolated [3]. Ectopic prostatic tissue has histological and immunohistochemical characteristics that are indistinguishable from those of normal prostatic tissue, and most likely represents the persistence of embryonic structures [1].

Making a preoperative diagnosis of ectopic prostatic tissue in the retrovesical space is extremely difficult. The majority of previously reported similar cases were diagnosed postoperatively. Transrectal ultrasound-guided biopsy was performed only in 3 previous cases [4,12]. In the present case, we also performed a needle biopsy but didn't get the retrovesical tumor; instead, the unexpected prostatic cancer was found. Thus, in clinical practice when a tumor is found in the retrovesical space of man, and the differential diagnosis included ectopic prostate, prostatic utricle cyst, prostatic abscess, seminal vesicle hydrops/cyst or empyema, large ectopic ureterocele and sarcoma [13]. And if elevated PSA levels is also detected in the patient, there are at least three possibilities should be thought of, including elevated PSA caused by ectopic prostate itself, malignant changes in ectopic prostate, and ectopic prostate with primary prostate cancer like our case.

## Conclusion

In conclusion, this is an interesting case which has not been reported previously. The presence of retrovesical ectopic prostate accompanied by primary prostate cancer is a relatively rare finding and may reflect divergent differentiation or a malformative process.

## Consent

Written informed consent was obtained from the patient for publication of this report and any accompanying images.

## Abbreviation

PSA: Prostate-specific antigen.

## Competing interests

The authors declare that they have no competing interests.

## Authors’ contributions

FQT, XX drafted the manuscript. JQ, BHS, KS, QHY and DSS also assisted with manuscript preparation. XYZ revised the manuscript. All authors have read and approved the final manuscript.
